# Diagnostic Performance of Metagenomic Next-Generation Sequencing (mNGS) and Culture in Infected Pancreatic Necrosis: A Systematic Review and Meta-Analysis

**DOI:** 10.1007/s10620-025-09474-1

**Published:** 2025-10-18

**Authors:** Aizaz Ali, Asad Iqbal Khattak, Dikshit Chawla, Fariha Hasan, Hafsa Khan, Muhammad Abdullah Ali, Abdul Moeez, Sana Tanveer, Ubaidullah, Muhammad Jibran Afridi, Jibran Ikram, ⁠Saad Hayat, Fatima Zehra Shah, Ahmed Nadeem, Muhammad Ahmad Nadeem, Ali Mushtaq, Waqqas Haroon, Hareesha Rishab Bharadwaj, Dushyant Singh Dahiya, Emad Mansoor

**Affiliations:** 1 Khyber Medical College, Peshawar, Pakistan; 2 Bacha Khan Medical College, Mardan, Pakistan; 3 Government Medical College, Patiala, India; 4 Cooper University Hospital, New jersy, USA; 5 Khyber Girls Medical College, Peshawar, Pakistan; 6 Cleveland Clinic Foundation, Cleveland, OH USA; 7 Khyber College of Dentistry, Peshawar, Pakistan; 8 Department of Liver Transplant Surgery, Digestive Diseases and Surgery Institute, Cleveland Clinic, Cleveland, OH USA; 9 Faculty of Biology Medicine and Health, University of Manchester, Manchester, UK; 10 Department of Gastroenterology and Hepatology, School of Medicine, University of Kansas, Kansas, USA; 11 Case Western Reserve University/University Hospitals Cleveland Medical Center/Louis Stokes Cleveland VA Medical Center, Cleveland, OH USA

**Keywords:** Metagenomic next-generation sequencing, Infected pancreatic necrosis, Diagnostic, Acute pancreatitis

## Abstract

**Background:**

Infected pancreatic necrosis (IPN) is a severe complication of acute pancreatitis, requiring prompt diagnosis. Conventional microbial culture, the current gold standard, has limitations in sensitivity and turnaround time. Metagenomic next-generation sequencing (mNGS) offers rapid, comprehensive pathogen detection, but its diagnostic performance for IPN remains unclear.

**Methods:**

We conducted a systematic review and meta-analysis following PRISMA-DTA guidelines, prospectively registered in PROSPERO (CRD420251008574). PubMed, Embase, and Web of Science databases were searched from inception to March 2025. Seven studies (313 patients) evaluating mNGS for IPN diagnosis were included, with four providing direct comparisons to culture. Pooled sensitivity, specificity, and area under the curve (AUC) were calculated using a random-effects model. Heterogeneity was assessed using I^2^ statistics.

**Results:**

In double-arm analysis, mNGS showed significantly higher sensitivity (0.87, 95% CI: 0.72–0.95) than culture (0.36, 95% CI: 0.23–0.51), with comparable specificity (0.83 for both). The AUC for mNGS (0.92, 95% CI: 0.79–0.94) surpassed that of culture (0.52, 95% CI: 0.27–0.86). Single-arm analysis confirmed mNGS as a reliable standalone test (sensitivity: 0.86; specificity: 0.85; AUC: 0.89). A threshold effect (r = − 0.991) indicated variability in diagnostic criteria across studies.

**Conclusions:**

mNGS outperforms culture in diagnosing IPN, offering higher sensitivity and faster results. Its ability to detect diverse pathogens, including fastidious and polymicrobial infections, makes it a valuable tool for early intervention. However, challenges like cost, standardization, and interpretation persist. Future studies should focus on prospective validation and cost-effectiveness to integrate mNGS into routine clinical practice.

**Supplementary Information:**

The online version contains supplementary material available at 10.1007/s10620-025-09474-1.

## Introduction

Acute pancreatitis (AP) is one of the most prevalent gastrointestinal disorders worldwide [1]. Patients with mild, self-limiting AP are typically discharged without complications. However, approximately, 20% of AP patients develop a complicated and prolonged clinical course when infected pancreatic necrosis (IPN) occurs [2, 3]. Early diagnosis and prompt intervention are crucial for improving patient outcomes. Despite the availability of various diagnostic techniques and predictors, such as blood cultures, the “air bubble sign” in necrotic collections, serum inflammatory markers (e.g., procalcitonin and C–reactive protein), and fine-needle aspiration (FNA) for bacteriology, early detection of IPN remains challenging [4]. The current gold standard, conventional microbial culture, requires at least 48 h to yield results and has suboptimal sensitivity [5].

An alternative or complementary approach to traditional culture-based methods is metagenomic next-generation sequencing (mNGS). This emerging technology is increasingly used for the clinical diagnosis of infectious diseases, particularly those that are rare, difficult to detect, novel, or severe [6]. By directly analyzing the genetic composition and functional characteristics of microorganisms, including bacteria, viruses, fungi, atypical pathogens, and parasites, mNGS can identify known and novel pathogens. Its high sensitivity and rapid turnaround time make it a valuable diagnostic tool [7, 8].

Additionally, mNGS offers the advantage of detecting potential antibiotic resistance genes, aiding in treatment selection, while semi-quantitatively assessing microbial load through sequence read counts [9, 10]. Currently, mNGS is widely employed in diagnosing and managing various infections, including localized abscesses, bone and joint infections, unexplained pneumonia, and central nervous system infections [8, 11–13]. However, its role in diagnosing IPN has yet to be fully explored. This is the first meta-analysis we perform, comparing mNGS versus culture for the diagnosis of IPN.

## Methods

### Study Design and Protocol Registration

This meta-analysis was completed according to the guidelines described by Preferred Reporting Items for Systematic Review and Meta-analysis of Diagnostic Test Accuracy studies (PRISMA-DTA) [14, 15]. The protocol was registered prospectively with the international prospective register of systematic reviews (PROSPERO: CRD420251008574).

### Search Strategy and Screening

Systematic searches of electronic databases PubMed, Embase, and Web of Science were conducted from inception to March 2025 using the following terms: “metagenomic next-generation sequencing,” “mNGS,” “and Infected pancreatic necrosis,” “IPN.” A detailed search strategy is outlined in Supplementary Table 1. No language filters were applied to our search. Two authors (A.A. and A.I.) independently screened titles and abstracts and evaluated the articles for eligibility based on pre-specified criteria.

### Eligibility Criteria

Studies were eligible if (1) they included adult patients with suspected infected pancreatic necrosis, (2) they provided data on diagnostic test accuracy for mNGS, (3) they were randomized controlled trials (RCTs), cohort studies, and cross-sectional studies, and (4) they are in any language. Studies were excluded if they (1) were confirmed cases of infected pancreatic necrosis or extra pancreatic complications, (2) presented data on the pediatric population, (3) were abstracts, case reports, or case series, and (4) did not provide sufficient data for the confusion matrix of diagnostic tests (true positives, false positives, false negatives, and true negatives).

#### Data Extraction

Data were extracted by two authors (A.I. and D.C.) into the excel sheet independently. mNGS was considered the index test, while conventional microbial culture served as the reference standard for evaluating the diagnostic accuracy of mNGS. For the calculation of the diagnostic accuracy of culture itself, the included studies used the final clinical diagnosis (based on microbiological evidence, imaging findings, and clinical course) as the gold standard. Data for true positives, false positives, false negatives, and true negatives were extracted accordingly. For each included study, values for true positives, false positives, false negatives, and true negatives were extracted. When these values were not explicitly reported, they were calculated using the following parameters: sample size, prevalence, sensitivity, and specificity as reported for mNGS and culture, applying epidemiological formulas [16]. We also attempted to extract technical details of the NGS methods, including manufacturer/platform and cutoff values. However, none of the included studies reported this information.

#### Statistical Analysis

The primary outcomes were the sensitivity and specificity of mNGS and culture. Pooled sensitivities, specificities, and 95% confidence intervals (CIs) were calculated using a generalized linear mixed model (GLMM; univariate random-effects), and the results were displayed as forest plots. For summary receiver operating characteristic (SROC) curves and the estimation of the area under the curve (AUC), a bivariate random-effects model was applied. Heterogeneity was assessed using Cochrane’s Q test and Higgins and Thompson’s I² statistics, with I² values of < 25%, < 50%, and > 50% indicating low, moderate, and substantial heterogeneity, respectively [17]. SROC curves and AUC were derived using R (version 4.4.2, 2024–10-31, UCRT). The “mada” package of R software (R Foundation for Statistical Computing, Vienna, Austria) was used for all analyses. Publication bias was assessed using funnel plot asymmetry, visually inspected for evidence of small-study effects. Statistical evaluation was performed using Egger’s regression test for funnel plot asymmetry [18] and Begg’s rank correlation test [19]. A p value of < 0.05 was considered statistically significant for both tests.

#### Quality and GRADE Assessment

The quality of the included studies was independently assessed by two reviewers (A.I. and A.A.) using the revised Quality Assessment of Diagnostic Accuracy Studies-2 (QUADAS-2) tool [20] and GRADEpro to evaluate the certainty of our findings, following the Grading of Recommendations, Assessment, Development, and Evaluation (GRADE) approach [21].

## Results

**Fig. 1:**
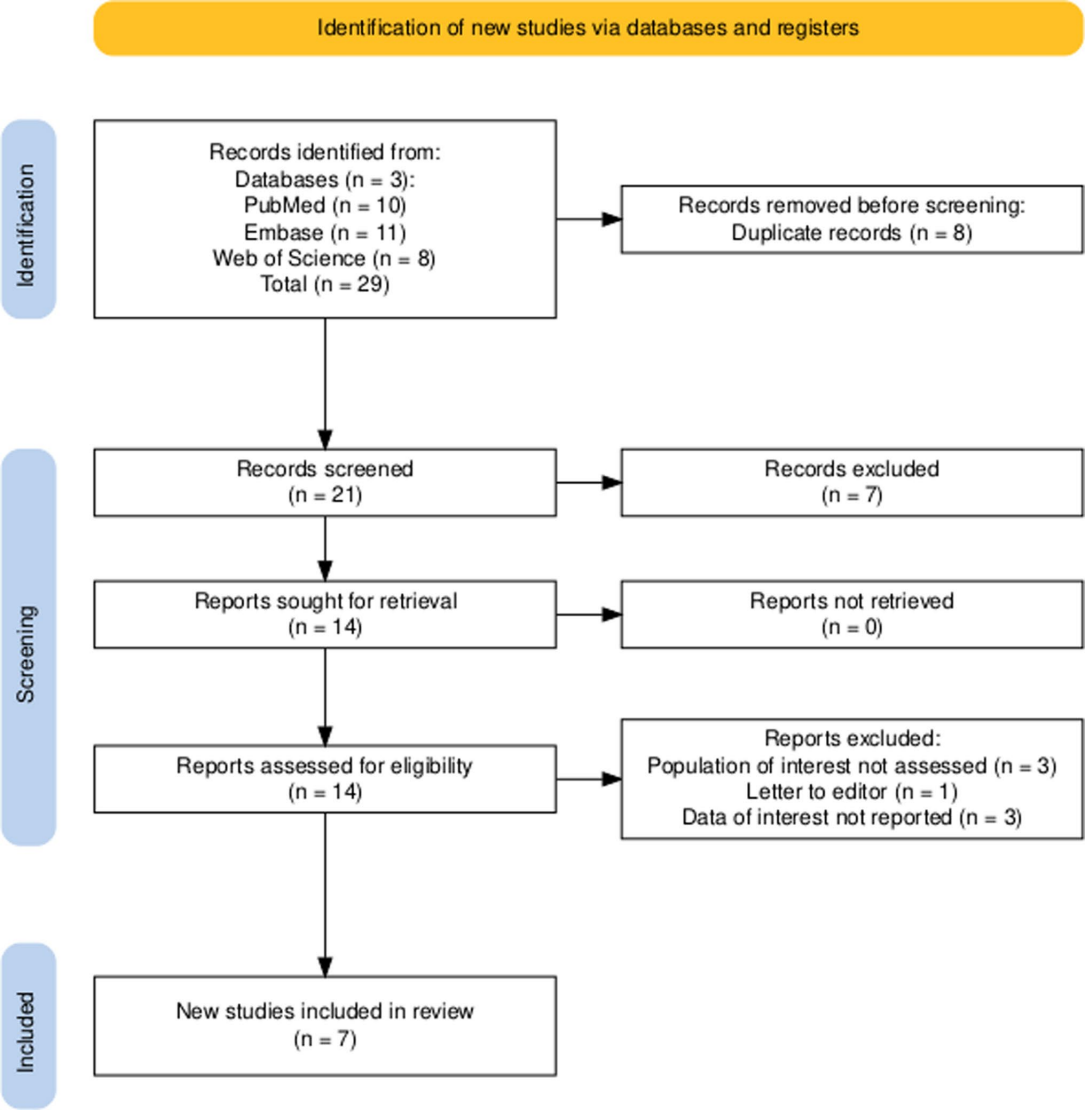
Prisma Flowchart showing study selection process using primary screening and secondary screening

The initial search yielded 29 results. After removing duplicate and ineligible studies, 14 remained and were thoroughly reviewed based on the inclusion criteria. Of these, a total of 7 studies [22–27] [28] were included, comprising 313 patients Fig. 1. Among these, four studies provided direct comparisons between mNGS and culture (double-arm), while three were single-arm evaluations of mNGS. The general characteristics of the included studies are presented in Table 1. The assessment outcomes of each study, as determined by QUADAS-2, are listed in Supplementary Fig. 1.

**Table 1:** Characteristics of studies evaluating mNGS (metagenomic next-generation sequencing) and culture for diagnosing infected pancreatic necrosis (IPN) in acute pancreatitis

Author	Year	Country	Sample type	Diagnostic test	Sample size (n)	TP	FP	FN	TN	Study type	Language	Turnaround Time (mNGS /Culture[median hours])
Lin (25) 2024 China Blood				mNGS	70	26	6	4	34	Prospective Cohort	English	Not Reported
				Culture		8	5	22	35			
Lin (24) 2022 China			Blood	mNGS	45	20	7	1	14	Prospective Cohort	English	37.7 /115.2 (mean. Hours)
				Culture		5	9	16	12			
			Peripancreatic fluid	mNGS	24	11	4	1	8			
Hong (23)	2022	China	Pancreatic fluid	mNGS	40	22	0	3	15	Retrospective Cohort	English	42/60
Hong (22)	2022	China	Plasma	mNGS	44	20	4	3	17	Retrospective Cohort	English	46.50/73.50
Hong (21)	2024	China	Peripancreatic fluid	mNGS	20	14	1	3	2	Prospective Cohort	English	43/120
Zhang (20)	2024	China	Pancreatic fluid	mNGS	45	32	1	13	20	Retrospective Cohort	English	25.5/88
				culture		14	18	11	2			
				mNGS	25	16	2	1	6	Prospective Cohort	Chinese	43/111
				Culture		6	2	11	6			

*FN* false negative, *FP* false positive, *mNGS* metagenomic next-generation sequencing, *TN* true negative *TP* true positive, *n* number of patients

### Double-Arm Analysis

**Fig. 2:**
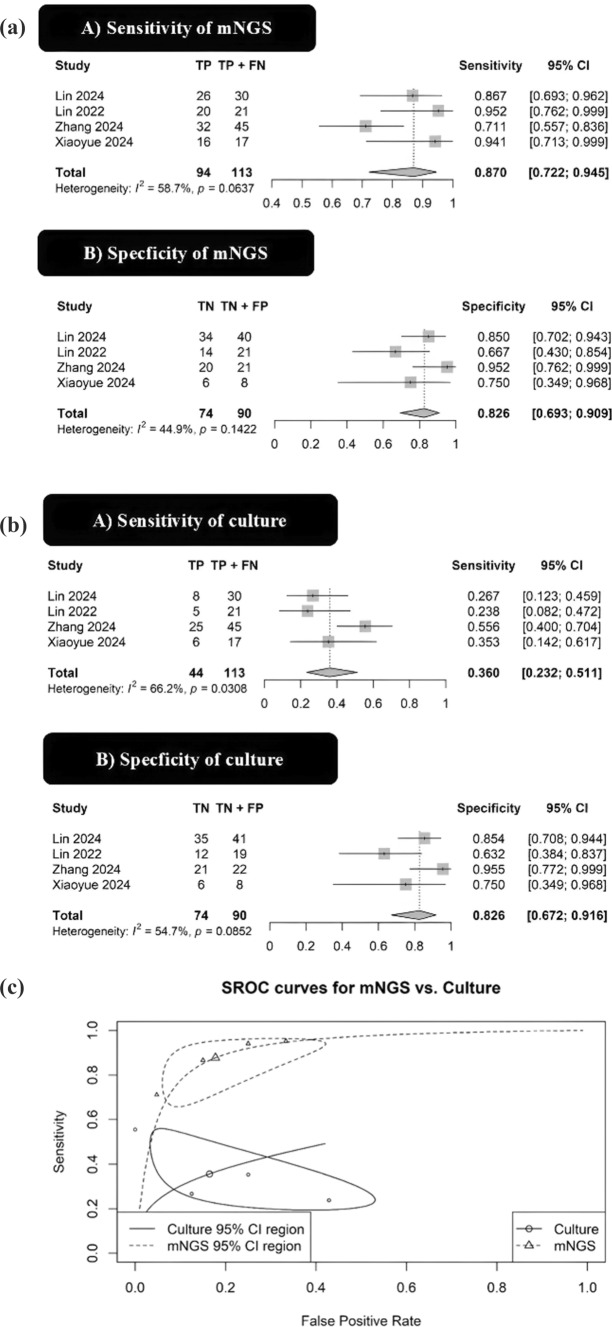
Forest plots summarizing the (**A**) sensitivity, (**B**) specificity of metagenomic next-generation sequencing (mNGS) for diagnosing infected pancreatic necrosis. Pooled estimates were calculated using a random-effects model. Squares represent individual study estimates; diamonds represent pooled estimates with 95% confidence intervals (CI). *I*^2^ quantifies heterogeneity. *CI* confidence interval, *FN* false negative, *FP* false positive; *mNGS* metagenomic next-generation sequencing, *TN* true negative, *TP* true positive. **B **Forest plots summarizing the (**A**) sensitivity, (**B**) specificity of culture for diagnosing infected pancreatic necrosis. Pooled estimates were calculated using a random-effects model. Squares represent individual study estimates; diamonds represent pooled estimates with 95% confidence intervals (CI). *I*^2^ quantifies heterogeneity. The substantial heterogeneity in sensitivity (*I*^2^ = 66.2%) is likely attributable to the sample type**.** The study by Zhang et al. (2024), which utilized pancreatic fluid, reported a higher sensitivity than the three studies that relied on blood samples. *CI* confidence interval, *FN* false negative, *FP* false positive, *TN* true negative, *TP* true positive. **C** Summary receiver operating characteristic (sROC) curves comparing the diagnostic accuracy of metagenomic next-generation sequencing (mNGS) and culture. The AUC for mNGS (0.92, 95% CI: 0.79—0.94) was significantly greater than for culture (0.52, 95% CI: 0.27–0.87), with a difference in AUC of 0.40 (95% CI: 0.02 –0.62; *P* = 0.036). The strong negative correlation (*r* = − 0.99) between logit-transformed sensitivity and specificity for mNGS indicates a significant threshold effect. Ellipses represent the 95% confidence regions for each test. *AUC* area under the curve, *CI* confidence interval, *mNGS* metagenomic next-generation sequencing, *sROC* summary receiver operating characteristic

A total of four studies directly comparing the diagnostic accuracy of mNGS and conventional culture were included in this meta-analysis. mNGS demonstrated a pooled sensitivity of 0.87 (95% CI: 0.72–0.95) and specificity of 0.83 (95% CI: 0.69–0.91), yielding an AUC of 0.92 (95% CI: 0.79–0.94) (Fig. 2A). In contrast, culture exhibited a pooled sensitivity of 0.36 (95% CI: 0.23–0.51) and specificity of 0.83 (95% CI: 0.67–0.92), with an AUC of 0.52 (95% CI: 0.27–0.86) (Fig. 2B). The difference in AUC between mNGS and culture was 0.40 (95% CI: 0.02—0.62; *P* = 0.036), indicating a significantly superior diagnostic performance of mNGS compared to culture. The correlation coefficient between logitsens and logitspec, *r* = − 0.991, shows a significant threshold effect. The diagnostic performance of the four studies that reported data for both mNGS and culture is given in Supplementary Table 2.

#### Single-Arm Analysis

**Fig. 3:**
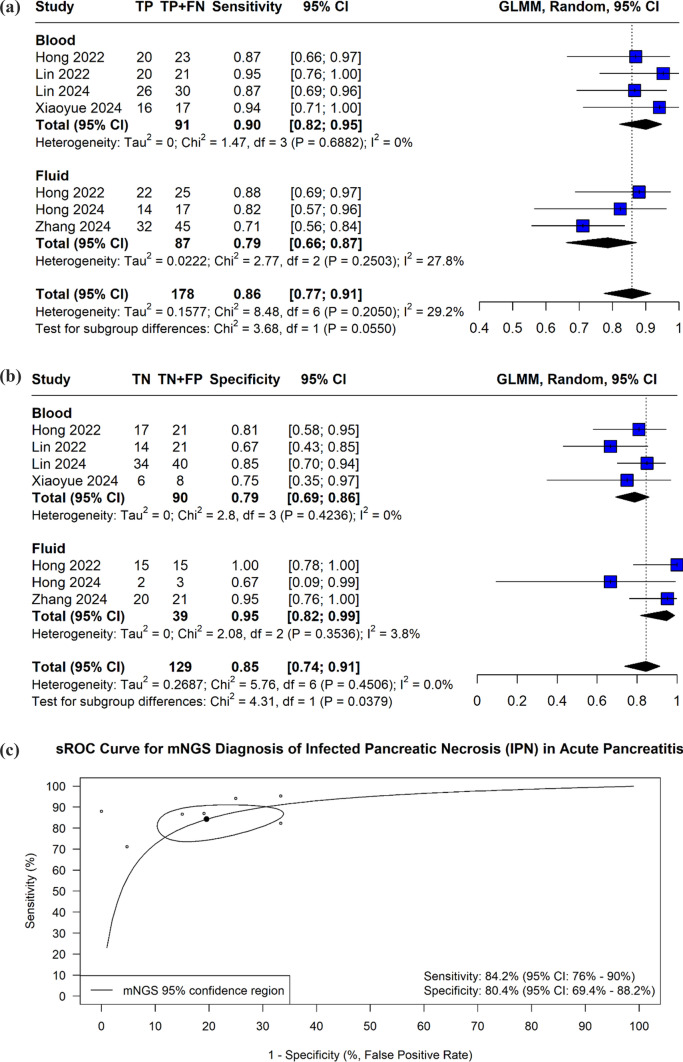
Forest plot of the pooled sensitivity of mNGS for diagnosing infected pancreatic necrosis, stratified by sample type**.** Estimates were generated using a generalized linear mixed model (GLMM) with random effects. Squares represent individual study estimates with 95% Cis; diamonds represent pooled subgroup and overall estimates. *I*^2^ quantifies heterogeneity, *CI* confidence interval, *mNGS* metagenomic next-generation sequencing. **B**Forest plot of the pooled specificity of mNGS for diagnosing infected pancreatic necrosis, stratified by sample type. **C **Summary receiver operating characteristic (sROC) curve for mNGS diagnostic performance The solid square marks the summary operating point (sensitivity: 84.2%; specificity: 80.4%). The area under the curve (AUC) was 0.89 (95% CI: 0.78—0.90). The solid curve is the sROC curve, and the dashed ellipse represents the 95% confidence region. The dotted line indicates no discriminatory ability (*AUC* = 0.5). Pooled estimates were derived using a bivariate random-effects model. Slight differences are observed when compared to univariate pooling due to the bivariate model’s accounting for the negative correlation between sensitivity and specificity. *AUC* area under the curve, *CI* confidence interval, *mNGS* metagenomic next-generation sequencing *sROC* summary receiver operating characteristic

Single-arm analysis of mNGS as a standalone diagnostic modality was also performed. Bivariate analysis demonstrated a pooled sensitivity of 0.86 (95% CI: 0.77–0.91) and specificity of 0.85 (95% CI: 0.74–0.91), yielding an AUC of 0.89 (95% CI: 0.78–0.90) Fig. 3A, B, and C. Diagnostic performance of seven studies reporting data for mNGS only is given in Supplementary Table 3.

#### Publication Bias and Quality Assessment

Review of the funnel plot could not rule out the potential for publication bias for mNSG and culture to diagnose IPN in pancreatitis (Supplementary Fig. 3, 4). Despite some concerns in specific domains, the overall quality of the studies was satisfactory, supporting the reliability of the findings in this meta-analysis (Supplementary Fig. 1). The certainty of evidence, assessed using the GRADE framework, was high for both true positive and true negative estimates, reinforcing the reliability of these findings (Supplementary Table 4).

## Discussion

In this systematic review and diagnostic meta-analysis comprising 7 studies and 313 patients, we compared metagenomic next-generation sequencing (mNGS) and standard culture techniques for the diagnosis of infected pancreatic necrosis (IPN) in patients with pancreatitis. Both double-arm and single-arm analyses were conducted. Our findings demonstrated superior diagnostic performance of mNGS over culture, with higher sensitivity, specificity, and area under the curve (AUC).

While our study focuses on IPN, similar diagnostic advantages of mNGS have been reported in other infectious conditions. For instance, a recent meta-analysis evaluating mNGS in cerebrospinal fluid samples from pediatric patients with central nervous system infections found a markedly improved detection rate compared to conventional methods [29]. Additionally, a case report on brain abscesses caused by oral pathogens highlighted that mNGS identified a broader range of organisms and offered higher sensitivity than culture [30]. These findings underscore the reliability of mNGS in detecting diverse pathogens, particularly in samples where traditional culture methods may be inadequate.

However, it is important to consider the established role of standard culture methods. Culture remains the clinical gold standard due to its accessibility, cost-effectiveness, and ability to provide live isolates for antimicrobial susceptibility testing. It allows definitive identification and resistance profiling of viable organisms, which is essential for tailoring antibiotic therapy in many settings. Nonetheless, culture is limited by its low sensitivity in polymicrobial infections, inability to detect fastidious or slow-growing organisms, and delayed turnaround time, which may be suboptimal in rapidly progressing infections like IPN.

In contrast, mNGS not only enhances diagnostic yield by detecting a wide range of bacterial, viral, fungal, and parasitic DNA in a single assay but also enables simultaneous antimicrobial resistance profiling and pathogen subtyping [31, 32]. This comprehensive approach supports precision medicine by facilitating targeted antimicrobial therapy and reducing time to appropriate treatment.

Despite its strengths, mNGS has limitations. It is currently more expensive and less widely available than culture, and the interpretation of its results can be challenging due to background noise from non-pathogenic organisms or host DNA. Furthermore, its clinical utility may be hindered by a lack of standardized protocols and limited reimbursement coverage in some healthcare settings. Some certain strengths of our study are the uniqueness of the study population chosen and the statistical analysis including both single-arm and double-arm analysis.

Our study also has specific limitations. Substantial heterogeneity was observed in some analyses (e.g., culture sensitivity I^2^ = 66.2%). Likely contributors include variability in sample type, timing of collection and prior antibiotic exposure, differences in sequencing platforms and bioinformatics pipelines, and variation in reference standards used across studies [7, 33]. Because key covariates were often not reported, formal meta-regression was not feasible [34, 35].

A threshold effect was evident in our data (negative correlation between sensitivity and specificity, r = − 0.991). This arises because different studies applied variable criteria for defining a positive result—for example, read-count or relative-abundance cutoffs in mNGS pipelines, or different clinical reference standards for infection [7, 33]. Such variability produces the expected trade-off between sensitivity and specificity across studies [34, 35]. Therefore, pooled values should be interpreted as averages across heterogeneous thresholds rather than fixed test characteristics. While mNGS consistently showed higher sensitivity than culture, its absolute performance in clinical practice will depend on local laboratory thresholds and validation procedures. This underscores the need for standardized reporting and harmonized diagnostic criteria to enhance generalizability [36].

A critical methodological consideration is the asymmetry in reference standards across the included studies, which may introduce bias in direct mNGS–culture comparisons [36]. In double-arm studies, mNGS was primarily benchmarked against conventional culture results (e.g., from blood or pancreatic fluid), an imperfect reference known for low sensitivity in polymicrobial or fastidious infections. In contrast, culture’s own accuracy was evaluated against a more comprehensive confirmatory IPN diagnosis, incorporating positive cultures from subsequent invasive procedures (e.g., drainage or necrosectomy), clinical progression (e.g., new-onset septic shock), and imaging. This broader standard could identify culture false negatives, potentially inflating mNGS’s apparent superiority (e.g., higher pooled sensitivity of 0.87 vs. 0.36). Single-arm studies similarly used confirmatory IPN as the reference for mNGS, often defining it via positive culture from necrotic specimens, creating circularity since culture is embedded in the gold standard. While this reflects real-world diagnostic challenges where no independent gold standard exists for IPN, it limits rigorous head-to-head comparisons. Future prospective studies should employ independent adjudication (e.g., blinded clinical panels excluding index test results) or standardized composites to mitigate this bias and validate mNGS’s clinical impact [37].

Additionally, as with most diagnostic test accuracy reviews, our analysis was limited to observational study designs (cross-sectional and cohort studies), which may introduce potential biases such as patient selection and heterogeneity in test interpretation. Another limitation of our review is that none of the included studies reported technical details such as the NGS manufacturer or cutoff values, which may impact reproducibility and cross-study comparisons. Furthermore, the limited data precluded comprehensive subgroup and sensitivity analyses. Future research should aim to address these gaps by conducting large-scale prospective studies that not only validate the diagnostic performance of mNGS but also directly compare its real-world impact alongside conventional culture methods [7].

## Supplementary Information

Below is the link to the electronic supplementary material.

Supplementary file1 (DOCX 3956 KB)

## Data Availability

All data, materials, and software applications support the published claims and comply with field standards.
