# Supplementary material for: Diagnostic Performance of Metagenomic Next-Generation Sequencing (mNGS) and Culture in Infected Pancreatic Necrosis: A Systematic Review and Meta-Analysis
Source: Dig Dis Sci. 2025 Oct 18;71(4):1323–31. doi: 10.1007/s10620-025-09474-1 (PMC13144204; doi:10.1007/s10620-025-09474-1)
Supplement: Supplementary file 1 — Supplementary file1 (DOCX 3956 KB) [file 10620_2025_9474_MOESM1_ESM.docx]

***SUPPLEMENTARY MATERIAL***

**Diagnostic Performance of Metagenomic Next-Generation Sequencing (mNGS) versus Culture in Infected Pancreatic Necrosis: A Systematic Review and Meta-Analysis**

**Table of content**

**Supplementary method 1.** Search strategy……………………………………………………………….2

**Supplementary Table 1.** Diagnostic performance of four studies reported data for both mNGS and culture……2

**Supplementary Table 2.** Diagnostic performance of eight studies reported data for mNGS only……….2

**Supplementary figure 1:** Risk of bias assessment of QUADAS-2 tool…………………………………..3

**Supplementary Figure 2:** Funnel plot of single arm analysis……………………………………………3

**Supplementary Figure 3:** Funnel plot of mNGS double arm analysis……………………………………4

**Supplementary figure 4:** Funnel plot of culture double arm analysis…………………………………….4

**Supplementary Table 3:** Grade assessment……………………………………………………………….5

**Table 1:** Search strategy:

| Search No | PubMed Query | Result |
| --- | --- | --- |
| #1 | Search: metagenomic next-generation sequencing Sort by: Most Recent | [6,900](https://pubmed.ncbi.nlm.nih.gov/?term=metagenomic+next-generation+sequencing&sort=date) |
| #2 | Search: mNGS Sort by: Most Recent | [2,133](https://pubmed.ncbi.nlm.nih.gov/?term=mNGS&sort=date) |
| #3 | Search: Infected pancreatic necrosis Sort by: Most Recent | [3,761](https://pubmed.ncbi.nlm.nih.gov/?term=Infected+pancreatic+necrosis&sort=date) |
| #4 | Search: IPN Sort by: Most Recent | [10,765](https://pubmed.ncbi.nlm.nih.gov/?term=IPN&sort=date) |
| #1 AND #2 | Search: (metagenomic next-generation sequencing) OR (mNGS) Sort by: Most Recent | [7,121](https://pubmed.ncbi.nlm.nih.gov/?term=%28metagenomic+next-generation+sequencing%29+OR+%28mNGS%29&sort=date) |
| #3 AND #4 | Search: (Infected pancreatic necrosis) OR (IPN) Sort by: Most Recent | [14,255](https://pubmed.ncbi.nlm.nih.gov/?term=%28Infected+pancreatic+necrosis%29+OR+%28IPN%29&sort=date) |
| #1 AND #2 AND #3 AND #4 | Search: ((metagenomic next-generation sequencing) OR (mNGS)) AND ((Infected pancreatic necrosis) OR (IPN)) Sort by: Most Recent | [10](https://pubmed.ncbi.nlm.nih.gov/?term=%28%28metagenomic+next-generation+sequencing%29+OR+%28mNGS%29%29+AND+%28%28Infected+pancreatic+necrosis%29+OR+%28IPN%29%29&sort=date) |
| Search No | Embase Query | Result |
| #1 | mngs | 2406 |
| #2 | ipn | 13981 |
| #3 | 'metagenomic next generation sequencing'/exp OR 'metagenomic next generation sequencing' | 2628 |
| #4 | 'infected pancreatic necrosis'/exp OR 'infected pancreatic necrosis' | 1211 |
| #5 | #1 AND #3 | 1948 |
| #6 | #2 AND #4 | 320 |
| #7 | #5 AND #6 | 11 |
| Search No | Web of Science Query | Result |
| #1 | ALL=((metagenomic next-generation sequencing) OR (mNGS)) | 4482 |
| #2 | ALL=((Infected pancreatic necrosis) OR (IPN)) | 49821 |
| #1 AND #2 | ALL=((metagenomic next-generation sequencing) OR (mNGS)) AND ALL=((Infected pancreatic necrosis) OR (IPN)) | 8 |

| **Parameters** | **mNGS** | **95% CI** | **culture** | **95% CI** |
| --- | --- | --- | --- | --- |
| No. of TP results | 94 | -- | 44 | -- |
| No. of TN results | 74 | -- | 74 | -- |
| No. of FP results | 16 | -- | 16 | -- |
| No. of FN results | 19 | -- | 69 | -- |
| Sensitivity (%) | 87 | 72-94 | 36 | 23-51 |
| Specificity (%) | 83 | 69-91 | 83 | 67-92 |
| AUC | 0.92 | 0.79-0.94 | 0.52 | 0.27-0.87 |
| **Supplementary Table 2. Diagnostic performance of four studies reported data for both mNGS and culture.** | | | | |

| **Parameters** | **mNGS** | **95% CI** |
| --- | --- | --- |
| No. of TP results | 161 | -- |
| No. of TN results | 116 | -- |
| No. of FP results | 25 | -- |
| No. of FN results | 29 | -- |
| Sensitivity (%) | 84.2 | 76-90 |
| Specficity (%) | 80.4 | 69.4-88.2 |
| AUC | 0.89 | 0.78 - 0.90 |
| **Supplementary Table 3. Diagnostic performance of seven studies reported data for mNGS only** | | |

**Supplementary figure 1:** Risk of bias assessment of QUADAS-2 tool.


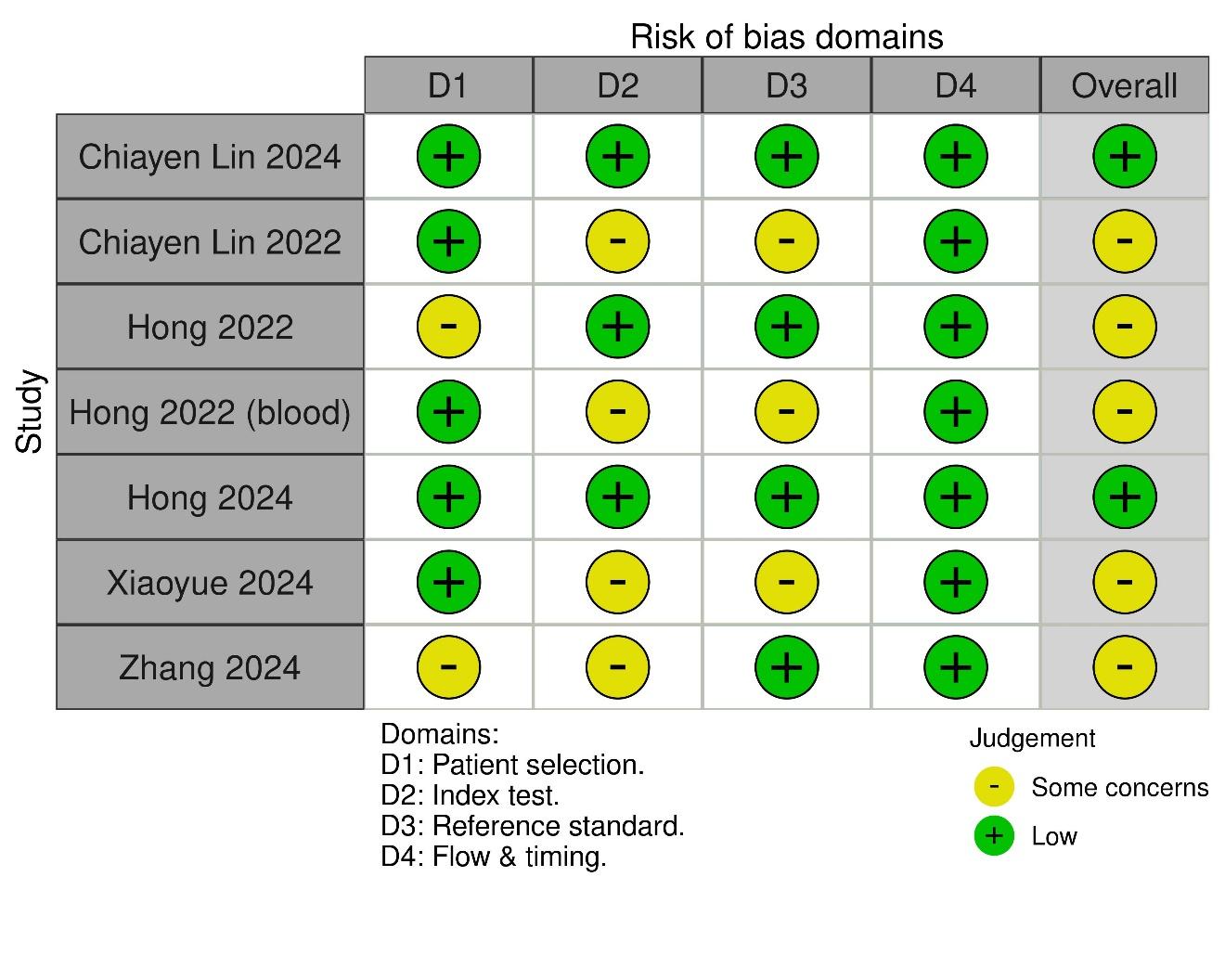


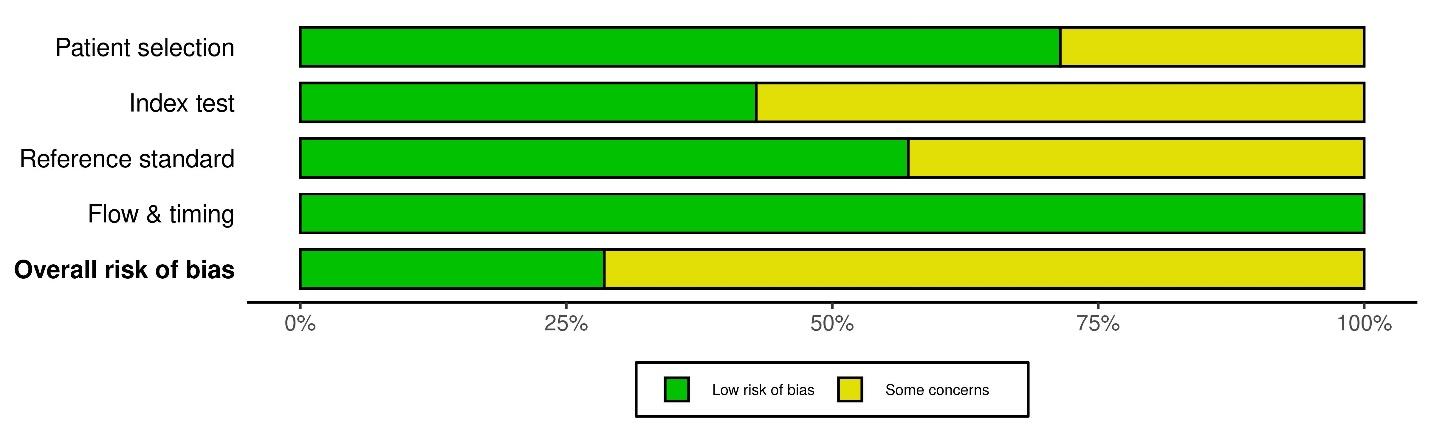


**Supplementary Figure 2: funnel plot of single-arm analysis.**


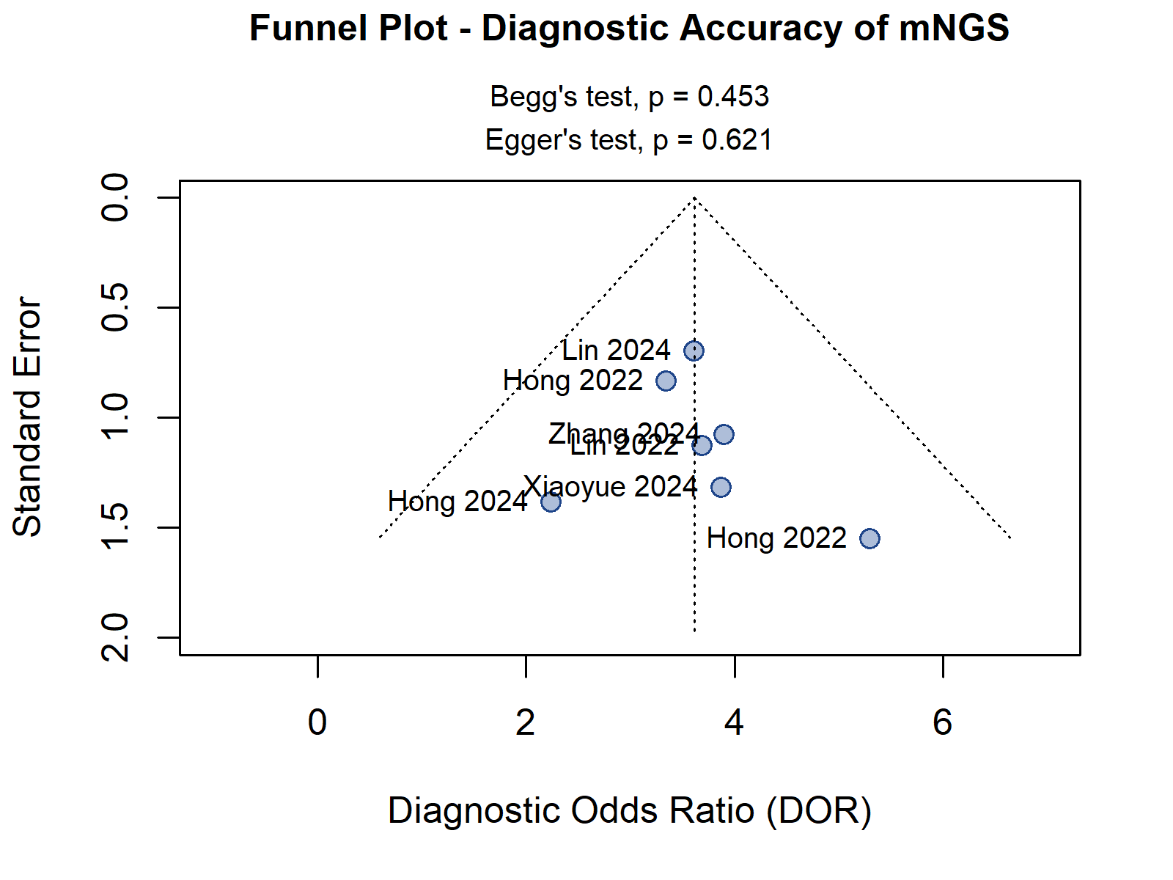


**Supplementary Figure 3: Funnel plot of mNGS double arm analysis.**

**
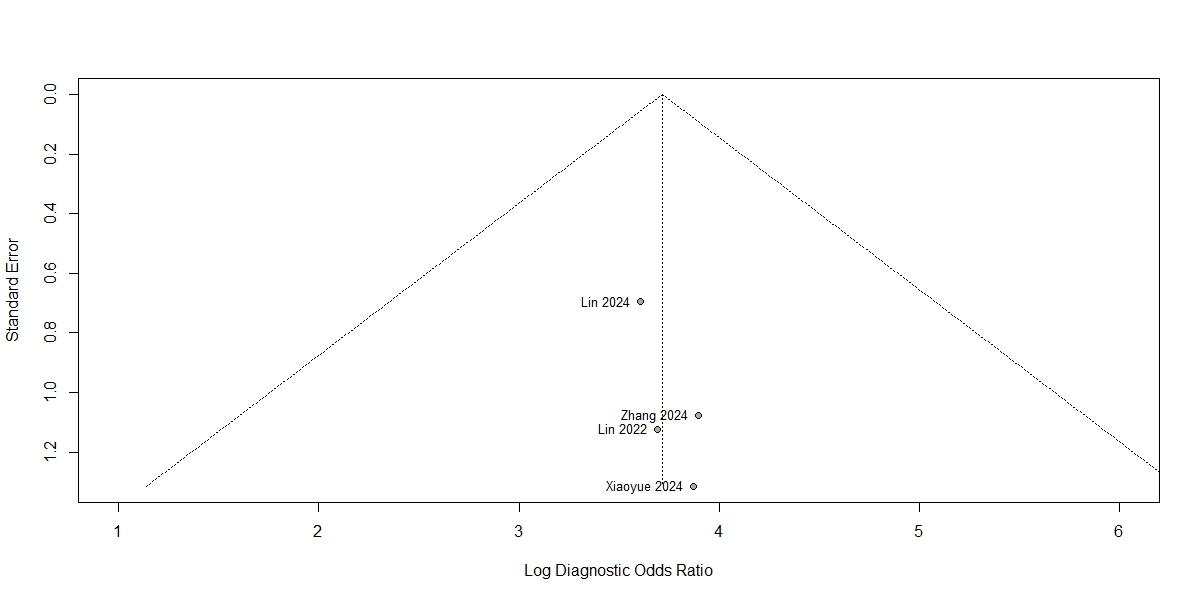
**

**Supplementary Figure 4: Funnel plot of culture double arm analysis.**


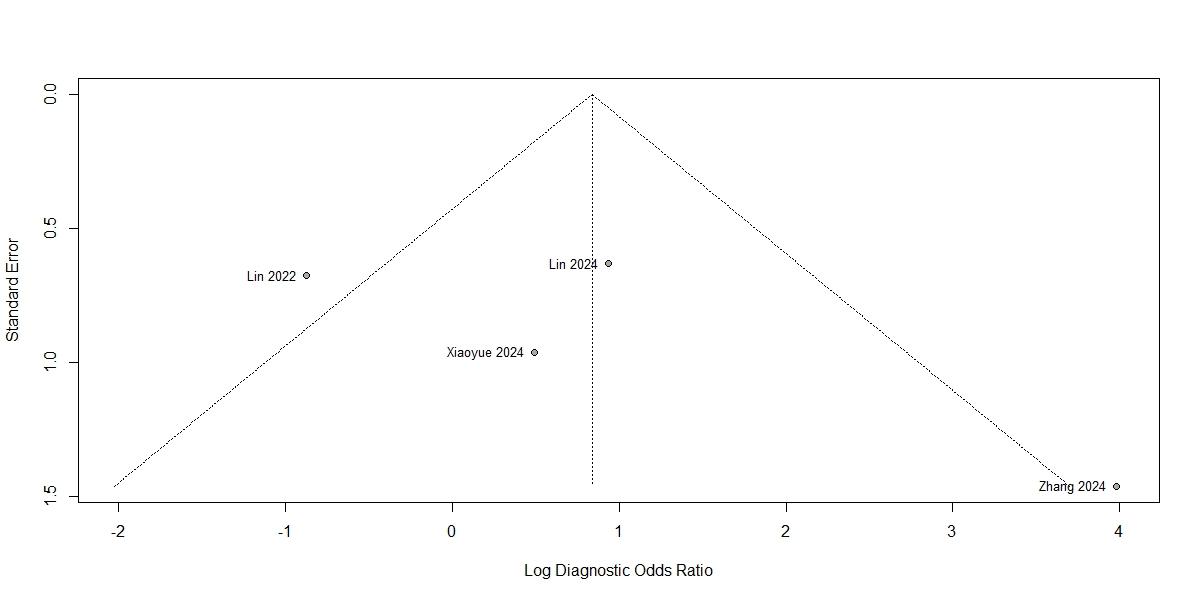


**Supplementary Table 4: Grade assessment**

**Should Metagenomic next-generation sequencing (mNGS) vs. Culture be used to diagnose Infected pancreatic necrosis (IPN) in Pancreatitis?**

**Patient or population**: Pancreatitis

**Setting**:

**New test**: Culture |**Cut-off value**:

**Reference test**: Culture |**Threshold**:

**Pooled sensitivity Metagenomic next-generation sequencing (mNGS)**:0.87 (95% CI: 0.72 to 0.95)|**Pooled specificity Metagenomic next-generation sequencing (mNGS)**:0.83 (95% CI: 0.69 to 0.91)

**Pooled sensitivity Culture**:0.36 (95% CI: 0.23 to 0.51)|**Pooled specificity Culture**:0.83 (95% CI: 0.67 to 0.92)

| **Test result** | **Number of results per 1,000 patients tested (95% CI)** | | **Number of participants  (studies)** | **Certainty of the Evidence (GRADE)** |
| --- | --- | --- | --- | --- |
|  | **Prevalence 0%**  Typically seen in | |  |  |
|  | Metagenomic next-generation sequencing (mNGS) | Culture |  |  |
| **True positives** | **0** (0 to 0) | **0** (0 to 0) | 113  (4) | ⨁⨁⨁⨁  **High** |
|  | **0 fewer TP in Metagenomic next-generation sequencing (mNGS)** | |  |  |
| **False negatives** | **0** (0 to 0) | **0** (0 to 0) |  |  |
|  | **0 fewer FN in Metagenomic next-generation sequencing (mNGS)** | |  |  |
| **True negatives** | **830** (690 to 910) | **830** (670 to 920) | 90  (4) | ⨁⨁⨁⨁  **High** |
|  | **0 fewer TN in Metagenomic next-generation sequencing (mNGS)** | |  |  |
| **False positives** | **170** (90 to 310) | **170** (80 to 330) |  |  |
|  | **0 fewer FP in Metagenomic next-generation sequencing (mNGS)** | |  |  |

**CI:** confidence interval
